# Uterine artery pseudoaneurysm after treatment of cesarean scar pregnancy: a case report

**DOI:** 10.1186/s12884-021-04166-w

**Published:** 2021-10-09

**Authors:** Jiao Wang, Qing Yang, Ningning Zhang, Dandan Wang

**Affiliations:** Department of Obstetrics and Gynecology, Shengjing Hospital of China Medical University, No.36 Sanhao Street, Heping District, Shenyang, 110004 P.R. China

**Keywords:** Uterus, Pseudoaneurysm, Cesarean scar pregnancy, Ultrasound, Uterine artery embolization

## Abstract

**Background:**

Pseudoaneurysms are formed when a local arterial wall ruptures, leading to hemorrhage and hematoma adjacent to the artery. Continuous perfusion of the injured artery increases the pressure in the lumen of the pseudoaneurysm. It may rupture and lead to massive hemorrhage that could be life-threatening. Cesarean scar pregnancy (CSP) is an ectopic pregnancy where the gestational sac is implanted in the cesarean scar. Uterine artery pseudoaneurysm (UAP) after CSP treatment is rare.

**Case presentation:**

We report the case of a 36-year-old Chinese woman who presented with acute massive vaginal bleeding 53 days after transabdominal scar pregnancy excision. Doppler ultrasound confirmed UAP. Selective uterine artery embolization (UAE) failed because of the thin and curved blood vessels. The lesion decreased in size after transvaginal ultrasound-guided direct thrombin injection (UGTI); however, massive vaginal bleeding recurred and endangered the patient’s life. The uterus was removed thereafter.

**Conclusions:**

UAP is a rare complication after CSP treatment that can lead to fatal massive hemorrhage. Ultrasound should be reexamined regularly after treatment of CSP. In case of unexplained vaginal bleeding, we should be alert to the existence of UAP and the possibility of rupture and take effective diagnosis and treatment measures promptly.

## Background

A pseudoaneurysm is formed when partial arterial wall deficiency leads to the accumulation of blood in the extra-luminal space [1]. Pseudoaneurysm of the uterine artery or its branches is usually a result of vascular trauma during invasive procedures such as cesarean section, vaginal delivery, myomectomy, hysterotomy, or dilation and curettage (D&C) [2]. Unlike true aneurysms that affect the intima, media, and adventitia layers, pseudoaneurysms usually only have a layer of loose connective tissue and communicates with the artery through its narrow neck. The turbulence makes the cavity larger and easier to rupture. Rupture of the uterine arterial pseudoaneurysm (UAP) can lead to massive hemorrhage that may endanger the woman’s reproductive health and may even be life-threatening. Cesarean scar pregnancy (CSP) refers to a pregnancy where fertilized ovum implants in the scar of a previous cesarean section. It is a type of ectopic pregnancy and is a long-term complication of cesarean section.

UAP is an uncommon cause of vaginal bleeding, especially after the treatment of CSP. In this report, we present the diagnosis and treatment of UAP in a patient with CSP after surgical treatment. Selective uterine artery embolization (UAE) and transvaginal ultrasound-guided direct thrombin injection (UGTI) failed, and the recurrence of life-threatening vaginal bleeding led to a hysterectomy.

## Case presentation

**Fig. 1:**
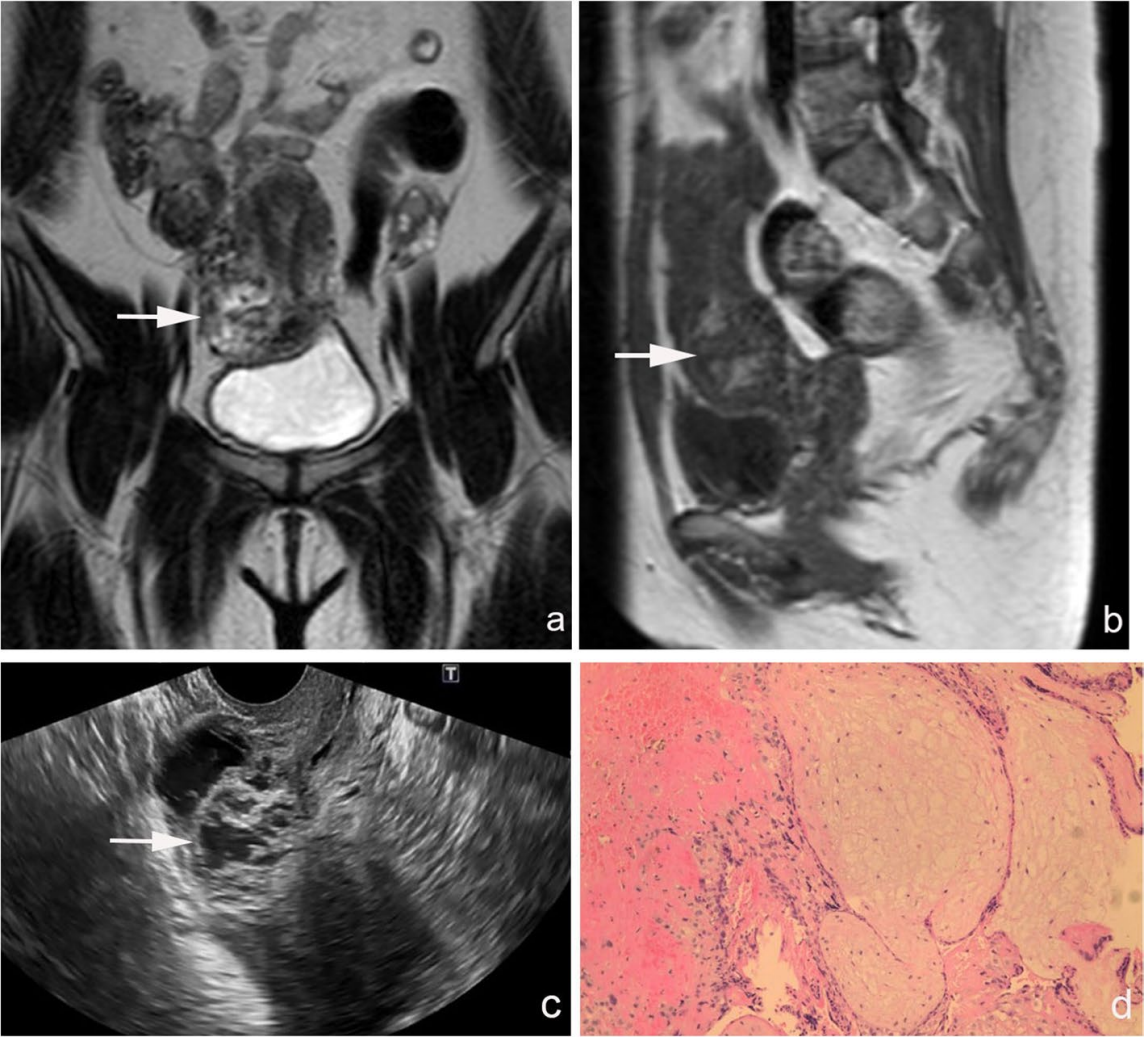
Ultrasound, MRI, and pathological images of cesarean scar pregnancy residue. (**a**, **b**) Pelvic MRI: the mass with abundant blood supply is seen in the isthmus of cervix; the size is about 4.8 cm × 4.1 cm × 3.7 cm. (**c**) TVUS: a 4.5 cm × 3.8 cm mass is in the cervical isthmus of the right anterior wall of the uterus, with a fuzzy boundary, protruding outside the uterus. (**d**) Under the microscope: degenerative villi in the hemorrhagic and necrotic tissues (hematoxylin and eosin: 100× magnification)

The patient is a 36-year-old female gravida 4, para 1 who had undergone cesarean section in 2010. She had undergone ultrasound-guided D&C due to CSP (gestational age: 6 weeks) 53 days prior (day 0) in a local hospital. There was a small amount of vaginal bleeding after the operation. On day 13, the serum human chorionic gonadotropin (hCG) level (16,846 mIU/mL) was higher than that before (day 4: 3103 mIU/mL), but no obvious abnormality was found by ultrasound in the local hospital. After taking mifepristone orally for 6 days, the serum hCG level continued to decrease. On day 39, the patient was reexamined and her serum hCG level was 892.9 mIU/mL. Doppler ultrasound showed a heterogeneous echo at the scar of the cesarean section, measuring 2.9 cm × 3.0 cm in size, which suggested a high possibility of residual pregnancy lesions. On day 48, the patient visited the outpatient department of our hospital. Color Doppler ultrasound showed a 4.5 cm × 3.8 cm mass in the cervical isthmus of the right anterior wall of the uterus, with a fuzzy boundary and abundant blood flow signal inside under color Doppler flow imaging (CDFI) scanning, protruding outside the uterus. Serum hCG level was 463.16 mIU/mL. Pelvic magnetic resonance imaging (MRI) showed the mass, approximately 4.8 cm × 4.1 cm × 3.7 cm, with an abundant blood supply, in the isthmus of the cervix (Fig. 1a, b). The hemoglobin level was 137 g/L. On day 53, the patient was admitted to the ward. After admission, ultrasonography showed that the mass increased to 5.3 cm × 5.3 cm × 4.2 cm (Fig. 1c). On day 55, transabdominal resection of the scar pregnancy lesion was performed. Intraoperative exploration showed extensive adhesion between the anterior wall of the uterus and bladder. After separation of the adhesion, an ultraviolet blue convex mass was seen on the right side of the cesarean scar, approximately 4 cm × 3 cm, with large blood vessels on the surface. The lesion was completely removed. Absorbable suture was used to repair the uterus. Intraoperative blood loss was about 1000 mL, and two units of red blood cell (RBC) suspension were transfused. The hemoglobin values of the patient were 110 g/L, 83 g/L and 86 g/L respectively on the first, fourth and sixth day after operation. Nine days after the operation (day 64), the patient recovered well with a small amount of vaginal bleeding; her hemoglobin level was 88 g/L, and serum hCG level decreased to 7.19 mIU/mL. Paraffin pathology showed degenerative villi in the hemorrhagic and necrotic tissues (Fig. 1d). The patient was discharged on day 64. Menstruation resumed 26 days after the operation (day 81).

**Fig. 2:**
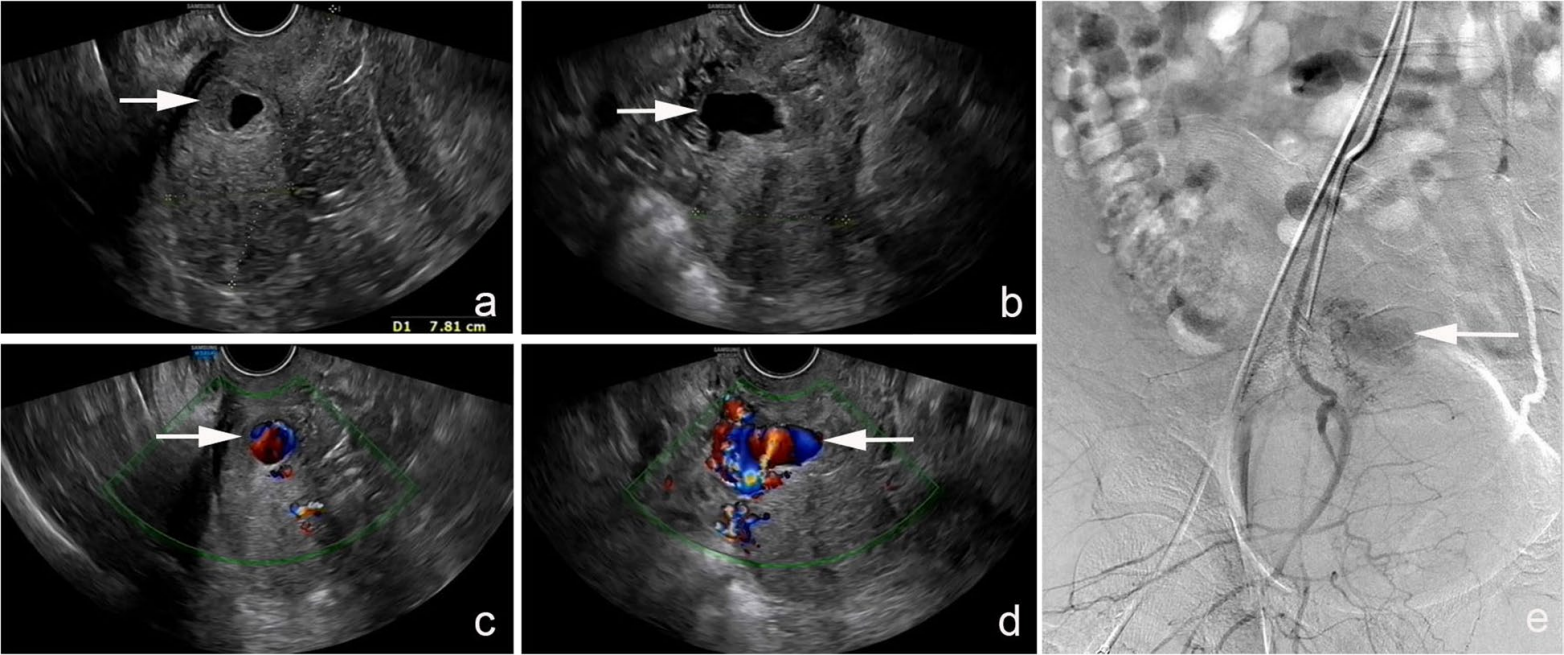
Doppler ultrasound and DSA images of uterine artery pseudoaneurysm. (**a**, **b**, **c**, **d**) TVUS: a 2.7 cm × 1.2 cm × 1.7 cm cystic mass is seen on the right side of the isthmus of cervix, with a clear boundary and irregular shape; the mass shows good perfusion. The low echo area with uneven thickness is seen around the liquid area, which is about 0.8 cm in the thicker area. (e) DSA: abnormal concentration of contrast medium is found

**Fig. 3:**
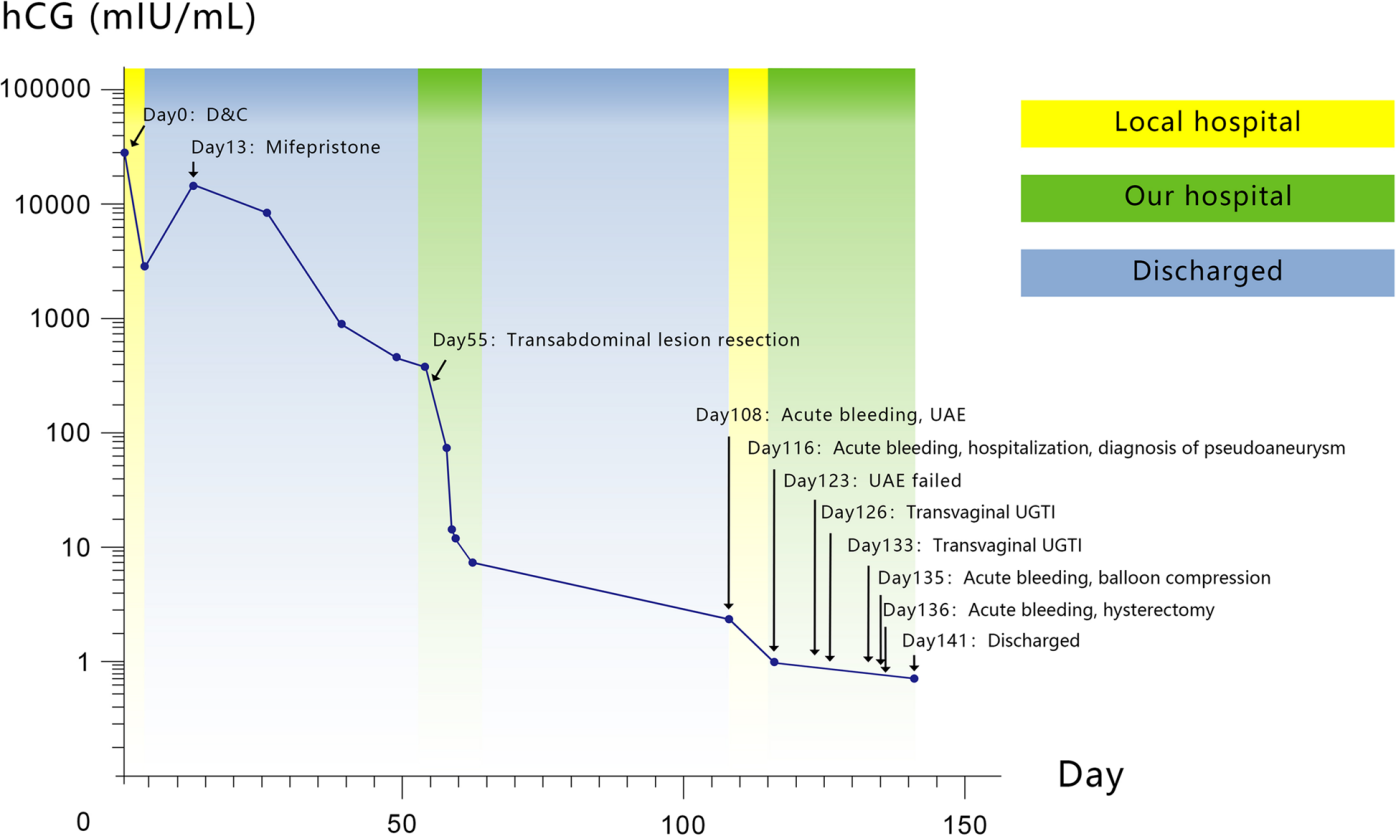
Serum hCG level and related treatment during the course of disease

Fifty-three days after the operation (day 108), the patient was readmitted to the local hospital due to massive vaginal bleeding, up to 10 times more than the amount of normal menstruation. She underwent emergent UAE to stop bleeding. Sixty-one days after the operation (day 116), massive vaginal bleeding occurred again, which was 5 times more than the amount of normal menstruation, so she revisited our hospital. Ultrasound showed a cystic mass measuring 2.7 cm × 1.2 cm × 1.7 cm on the right side of the isthmus of the cervix, with a clear boundary and irregular shape, and the mass had good perfusion. The local blood flow velocity was approximately 100 cm/s. A low echo area with uneven thickness was seen around the liquid area, which was about 0.8 cm in the thicker area (as shown in Fig. 2a, b, c, and d). A diagnosis of pseudoaneurysm with local thrombosis was made through ultrasound. Her hemoglobin level was 78 g/L. A repeat of the UAE was suggested. The patient initially refused; however, she agreed after several days of consideration. On day 123, uterine arteriography was performed under local anesthesia. After the catheter was inserted into the right internal iliac artery, an abnormal concentration of the contrast medium was found (Fig. 2e). A plan to precisely select the responsible vessel for vascular embolization was made. However, the approach failed because the target vessel was curved and slender. Next, transvaginal UGTI was performed on day 126. The surgery was successful, and there was a small amount of vaginal bleeding after the operation. On day 131, the lesion reduced to 1.2 cm × 1.2 cm in size. On day 133, transvaginal UGTI was performed again. On day 135, the patient had massive vaginal bleeding again, which was about 4 times more than the amount of normal menstruation. The patient then underwent intrauterine balloon compression. On day 136, the vaginal bleeding increased. A speculum examination showed a large number of clots and fresh blood flowing out of the vagina. Transabdominal hysterectomy was recommended because of the repeated failure of conservative treatment. The patient and her family agreed with the treatment regimen. On day 136, transabdominal hysterectomy was performed under general anesthesia. Intraoperative exploration showed a soft convex mass approximately 2 cm × 1 cm in size on the right side of the cesarean scar. The removed uterus was sent for pathological examination. The blood loss was approximately 200 mL, and two units of RBC suspension were infused. The patient recovered well after the operation and was discharged on day 141. The patient was followed up at 1 and 6 months postoperatively and there was no abnormality discovered. The entire treatment process is shown in Fig. 3.

## Discussion and conclusions

Pseudoaneurysms are caused by rupture of the arterial wall and localized hematoma in the surrounding soft tissue, which usually occur in superficial peripheral arteries but rarely in uterine arteries. UAP can be caused by obstetrics- and gynecology-related operations such as vaginal delivery, cesarean section, artificial abortion, myomectomy, and cervical conization. The most frequent cause of UAP is cesarean section [3]. There are also reports of UAP without a history of uterine operation [4]. CSP is a special ectopic pregnancy in which the gestational sac is implanted in the scar of cesarean section. The poor contractility of the scar tissue increases the risk of massive bleeding during the operation and may even lead to loss of fertility in serious cases. Recently, there has been an increase in the incidence of CSP. The patient in our case developed UAP after surgical treatment of CSP.

At present, the pathogenesis of UAP is not clear. Various operations might lead to inflammatory lesions and infection of the artery. Improper suturing techniques might cause local injury of the artery through which blood enters the uterine myometrial space; this could result in a localized hematoma and pseudoaneurysm. In our case, it is possible that the residual lesion of CSP had many blood vessels. The pseudoaneurysm might have been formed by inflammatory stimulation or improper suturing of the artery wall at the uterine incision after resection of the residual lesion of the CSP.

A true aneurysm is surrounded by three arterial layers of tunica intima, media, and adventitia, whereas in a pseudoaneurysm, boundaries are formed by a peripheral thrombus and perivascular tissues [5]. The arterial blood flow in a UAP forms turbulence in the cavity and hence a gradual increase in the size of the UAP. When the pressure in the UAP reaches a certain level, it may rupture and cause massive bleeding. When the pressure inside the cavity is released, the UAP can close by itself. The clinical manifestations of a UAP are non-specific and may be associated with a variety of factors, such as the cause of the disease, the location of the injured artery, and whether the cavity of UAP is connected with the uterine cavity or not. Some patients may even be asymptomatic, with the UAP found only on examination if the UAP cavity has thrombosed and closed by itself.

Angiography remains the gold standard for the diagnosis of UAP. Ultrasonography, computed tomography (CT), and MRI can also be used to assist diagnosis [5]. The increased CSP incidence rate and improvements in doctors’ understanding have improved the diagnosis of CSP. However, UAP is extremely rare after treatment of CSP. Ultrasound is one of the simplest and most important methods to diagnose UAP. In general, on grayscale ultrasonography, pseudoaneurysm has a characteristic sonographic appearance consisting of a pulsating anechoic or hypoechoic well defined cystic structure with or without associated pelvic hematoma or free fluid [6]. On color Doppler, the pseudoaneurysm systolic and diastolic flow creates a characteristic “to-and-fro” pattern, classically described as the “Yin-Yang” sign [7]; it is caused by changes of the pressure in the pseudoaneurysm and the blood vessel during the cardiac cycle. However, in practice, it is difficult to show the neck because the uterine artery is very small. In the gray-scale image, UAP may have sonographic manifestations similar to those of CSP, cystic changes in uterine fibroids, Nessler’s cyst of the cervix, and cyst of the uterine muscle wall, showing a complete hypoechoic area or liquid dark area. Therefore, color Doppler should be used to identify cystic structures in clinical practice. Meanwhile, a pseudoaneurysm should be distinguished from a uterine arteriovenous fistula (UAVF). In case of a UAVF, CDFI can show that blood in the artery enter the vein directly and continuously, with a high velocity and low resistance artery spectrum at the fistula. Digital subtraction angiography (DSA) is the gold standard for the diagnosis of UAP. It can directly display the blood flow in UAP and identify the artery that supplies it. When patients have active bleeding, contrast medium overflow can be observed, which can directly guide the embolization.

There is no standard for the treatment of CSP. The methods described for the treatment of CSP include hysteroscopy, laparoscopy, laparotomy, vaginal surgery, curettage, UAE, methotrexate (MTX), potassium chloride injection, needle guided balloon decompression, high intensity focused ultrasound imaging, and use of balloon catheter or a combination of these methods [8, 9]. The patient in our case underwent D&C in the local hospital first. After the operation, the patient visited our hospital because of suspected CSP residue. Because ultrasound and MRI showed exogenous CSP and a rich blood supply, we chose transabdominal lesion resection. However, a large amount of vaginal bleeding occurred 53 days after resection of the residual lesion, and UAP was confirmed by color Doppler ultrasound.

The treatment of UAP includes expectant management [10, 11], selective UAE, ligation of the internal iliac artery or uterine artery and its branches [12], intra-arterial catheter-directed thrombin injection [7], or percutaneous or transvaginal UGTI [13]; selective UAE is the first-line treatment. UAE is a safe treatment method with a high success rate and few reports of complications [14]. The specific treatment plan is guided by the size and location of the lesion, patient’s clinical manifestations, and resources of the hospital. Our patient had repeated massive vaginal bleeding. Before visiting our hospital, UAE was performed once in the local hospital. In our hospital, owing to the tortuous vascular anatomy, failure of arterial selection led to unsuccessful embolization. Thereafter, an ultrasound intervention was performed, and the pseudoaneurysm and vaginal bleeding reduced significantly in a short time. However, after two ultrasound-guided administrations of thrombin, a large amount of vaginal bleeding occurred, and the situation was critical. Intrauterine balloon compression had no significant effect. Finally, a transabdominal hysterectomy was performed.

We reviewed published literature and found two cases of UAP that developed after treatment of CSP (Table 1). One of the patients was a 25-year-old Chinese woman with a missed miscarriage in a CSP. A UAP measuring 7.1 cm × 4.4 cm × 3.9 cm was detected the next day after curettage by Doppler ultrasound. While the patient awaited admission to an advanced institution, the pseudoaneurysm ruptured spontaneously. The subsequent severe hemorrhage necessitated a hysterectomy [15]. A pseudoaneurysm was diagnosed by ultrasound in the other patient 16 days after local injection of MTX and potassium chloride with systemic MTX treatment. A large amount of vaginal bleeding occurred during D&C 21 days after the discovery. Emergency UAE was performed successfully to stop the bleeding [16]. Including our case, all three cases had a history of massive vaginal bleeding after treatment of CSP. However, the timing of the bleeding was different. Because the formation of UAP after treatment of CSP is extremely rare and there is no treatment guideline, we can only formulate an individualized follow-up plan according to the condition of the CSP patient. However, we still suggest that ultrasound should be reexamined regularly after CSP treatment (half a month, 1 month and 2 months after treatment), and serum hCG value should be monitored at the same time. In case of sudden massive vaginal bleeding, the possibility of the rupture of the UAP should be considered first. For patients with stable conditions, UAE is the first choice to preserve the fertility. Once fertility-preserving treatment methods fail and massive vaginal bleeding occurs and endangers patients’ lives, a hysterectomy should be performed.

**Table 1:** Characteristics of UAP after CSP reported in the literature

Authors	Year of publication	Age	Gravida	Para	Previous CS	Pregnancy weeks	Former intervention	Diagnostic methods	Feeding uterine artery	Management procedure
Mou et al. [15]	2014	25	2	1	1	8	D&C	Doppler ultrasound	Left	Hysterectomy
Kiyokawa et al. [16]	2018	37	2	1	1	6	Local MTX, systemic MTX	Doppler ultrasound, Enhanced MRI	Left	UAE
Our case		36	4	1	1	6	D&C, transabdominal CSP lesion resection	Doppler ultrasound	Right	UAE failed, transvaginal UGTI failed, hysterectomy

Abbreviations: *D&C* dilation and curettage; *MTX* methotrexate; *MRI* magnet resonance imaging; *CT* computed tomography; *UAE* uterine artery embolization; *UGTI* ultrasound-guided thrombin injection

There are some deficiencies in the diagnosis and treatment of our case. The patient suffered from sudden vaginal bleeding 53 days after resection of the residual lesion of CSP. A UAP was found after the ultrasound examination. For all the patients that we treat, we recommend that they return to our outpatient clinic for reexamination one month after the operation. However, this patient was not a local and did not follow our advice of timely follow-up. Hence, we were unable to monitor the patient’s postoperative situation. Timely follow-up would have helped identify the pseudoaneurysm earlier and ensure the provision of timely treatment, thus avoiding repeated vaginal bleeding and adverse consequences of hysterectomy.

UAP is a rare complication secondary to CSP, which can lead to fatal massive bleeding. Ultrasound should be reexamined regularly after treatment of CSP. In case of unexplained vaginal bleeding, we should be vigilant about the existence of UAP. We should consider the possibility of rupture so that we can take timely and effective treatment measures.

## Data Availability

The data obtained during the current study are available from the corresponding author on reasonable request.

## Abbreviations

CSP: Cesarean scar pregnancy
UAP: Uterine artery pseudoaneurysm
UAE: Uterine artery embolization
UGTI: Ultrasound-guided direct thrombin injection
D&C: Dilation and curettage
hCG: Human chorionic gonadotropin
CDFI: Color Doppler flow imaging
MRI: Magnetic resonance imaging
RBC: Red blood cell
CT: Computed tomography
UAVF: Uterine arteriovenous fistula
DSA: Digital subtraction angiography
MTX: Methotrexate
